# Breastfeeding in China: a review

**DOI:** 10.1186/1746-4358-4-6

**Published:** 2009-06-16

**Authors:** Fenglian Xu, Liqian Qiu, Colin W Binns, Xiaoxian Liu

**Affiliations:** 1 Medical College of Shihezi University; Xinjiang, PR China, 832002; 2 Women's Hospital, Zhejiang University, PR China, 310006; 3 School of Public Health and Curtin Health Innovation Research Institute, Curtin University of Technology, WA, Australia, 6845; 4 Tongji Medical College, Huazhong University of Science and Technology, PR China, 430013

## Abstract

This review aims to describe changes in breastfeeding and summarise the breastfeeding rates, duration and reasons of discontinuing 'any breastfeeding' or 'exclusive breastfeeding' in P.R. China. Breastfeeding rates in China fell during the 1970s when the use of breast milk substitutes became widespread, and reached the lowest point in the 1980s. As a result many efforts were introduced to promote breastfeeding. The breastfeeding rate in China started to increase in the 1990s, and since the mid-1990s 'any breastfeeding' rates in the majority of cities and provinces, including minority areas, have been above 80% at four months. But most cities and provinces did not reach the national target of 'exclusive breastfeeding' of 80%. The 'exclusive breastfeeding' rates in minority areas were relatively lower than comparable inland provinces. The mean duration of 'any breastfeeding' in the majority of cities or provinces was between seven and nine months. The common reasons for ceasing breastfeeding, or introducing water or other infant food before four months, were perceived breast milk insufficiency, mother going to work, maternal and child illness and breast problems. Incorrect traditional perceptions have a strong adverse influence on 'exclusive breastfeeding' in less developed areas or rural areas. China is a huge country, geographically and in population size, and there is considerable ethnic diversity. Therefore breastfeeding rates in different parts of China can vary considerably.

## Background

Breastfeeding is recognised as the normal method for feeding infants and is closely related to health during infancy and to chronic disease prevention in adulthood [1-3]. 'Exclusive breastfeeding' for the first six months of life and continued breastfeeding up to two years of age or beyond are recommended by WHO and other authorities[3,4]. The target set in the National Program of Action for Child Development in China in the 1990s was breastfeeding of 80% by 2000 (province based) and promoting 'exclusive breastfeeding' to four or six months[5]. The target was explained in many academic papers as 'exclusive breastfeeding' rate at four months of 80% by 2000 [6-8]. A new target set in the National Program of Action for Child Development in China in from 2001 to 2010 is a breastfeeding rate of 85% (province or municipality based) and timely introduction of complementary food[9]. However the type of breastfeeding is not specifically defined and the timeframe is not mentioned in the document.

With the promotion of breastfeeding becoming a national priority in China since the 1990s, a number of research studies on breastfeeding have been published. This review aims to describe breastfeeding and summarise breastfeeding rates, duration and reasons of discontinuing 'any breastfeeding' or 'exclusive breastfeeding' in P.R. China.

## Methods

Most of the studies on breastfeeding from China have been written in Chinese and very few in English. A literature search was undertaken using the PubMed, Science Direct and Proquest databases in the English language and the only major database available in Chinese, the Chinese Academic Journal Full Text Database (CNKI). The databases were searched using the key words: China, breastfeeding, breast feeding, breast-feeding and infant feeding. Where the abstract appeared relevant the full text paper was obtained. Since the Chinese database does not include papers earlier than 1990, the review was restricted to studies published since that time. However where data from periods earlier than 1990 were included in papers published since 1990, this has been included in the first part of the review. The review was restricted to the mainland provinces and autonomous regions of the Peoples Republic of China, and data from the regions and provinces of Macau, Hong Kong and Taiwan were not included.

### Definition of breastfeeding

The following definitions are used in this paper [10-14].

• Exclusive breastfeeding: Breastfeeding while giving no other food or liquid, not even water, with the exception of drops or syrups consisting of vitamins, mineral supplements or medicine;

• Full breastfeeding: Infant is breastfed and may also receive small amounts of culturally valued supplements – water, water-based drinks, fruit juice or ritualistic fluids;

• Partial breastfeeding: mixed feeding with breast milk and other sources of energy and nutrients;

• Any breastfeeding: The child has received breast milk with or without other drink, formula or other infant food;

• Breastfeeding: In some papers, 'breastfeeding' was not defined in the methods section and in these cases 'breastfeeding' was classified as "any breastfeeding" in this paper.

However it needs to be noted that in many papers only the term 'breastfeeding' has been used. This is interpreted in this review as 'any breastfeeding'.

## Results

### Transition of breastfeeding rates in China

**Figure 1:**
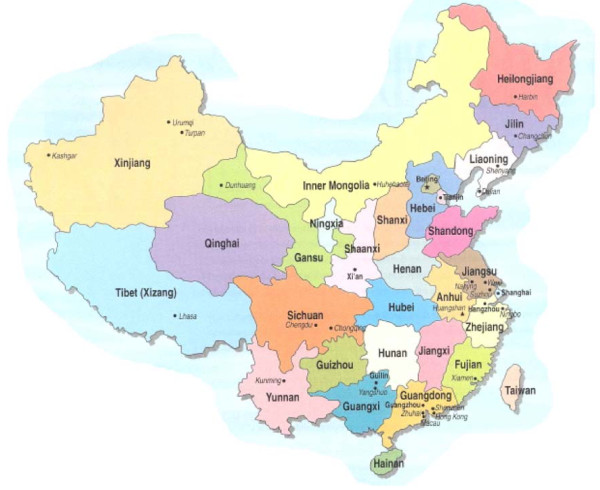
**The provinces and the major cities of PR China**.

There have been considerable changes in breastfeeding practices in China over the past forty years. The changes are reviewed in this section and details of samples and methods are shown in Tables 1 and 2. 'Ever breastfed' rates in both urban and rural areas were over 80% in the 1950s and 1960s [15]. But during the 1970s, the rates started to decline, especially in larger cities, when the use of breast milk substitutes became widespread [16]. See the attached map (Figure 1) for location of the major cities and provinces. In Beijing, as late as 1990, the breastfeeding rate remained at a low level. A cross sectional study in Beijing (n = 439) showed that 'any breastfeeding' rates at four months were 62.8%, 56.9%, 61.3% and 55.9% in 1989, 1990, 1991 and 1992 respectively and 'full breastfeeding' rates at four months were 35.3%, 29.3, 29.0%, and 31.5% respectively[7]. A retrospective study of 826 women in Tianjin, China (from Oct. 1981 to March 1982) showed that breastfeeding duration (breastfeeding was not defined in the methodology) was 25 months in 1932 and earlier, 24 months from 1933 to 1942, 20 months from 1943 to 1952, 17 months from 1953 to 1972, and 13 months from 1973 to 1982 [17].

**Table 1:** Breastfeeding rates (%) at four months from cohort studies, P.R. China

**Study details**	**Months of age**	**Exclusive breastfeeding**	**95%CI**		**Any breastfeeding**	**95%CI**	
Beijing [33] n = 100 Survey year: 1997	1	54.0	44.2	63.8	84.0	76.8	91.2
	2	46.0	36.2	55.8	80.0	72.2	87.8
	3	37.0	27.5	46.5	77.0	68.8	85.2
	4	37.0	27.5	46.5	76.0	67.6	84.4

Chongqing [34] n = 627 Survey year: 1999.	0^b^	80.5	77.4	83.6	93.5	91.6	95.4
	1	64.3	60.5	68.1	83.3	80.4	86.2
	2	52.3	48.4	56.2	71.6	68.1	75.1
	3	46.6	42.7	50.5	63.6	59.8	67.4
	4	42.9	39.0	46.8	59.2	55.4	63.0

Wuhan^a ^[35,36] n = 520 Survey year: 1994–1997.	0	89.2	86.5	91.9	97.7	96.4	99.0
	1	82.3	79.0	85.6	93.7	91.6	95.8
	2	70.6	66.7	74.5	87.4	84.5	90.3
	3	65.0	60.9	69.1	85.8	82.8	88.8
	4	55.6	51.3	59.9	84.0	80.8	87.2

Nanjing [37] n = 699 Survey year: 1995–1996	0	76.1	72.9	79.3	96.4	95.0	97.8
	1	65.5	61.8	69.2	94.2	92.4	96.0
	2	64.4	60.6	68.2	92.6	90.5	94.7
	3	65.9	61.7	70.1	90.4	87.8	93.0
	4	60.6	56.1	65.1	87.0	83.9	90.1

Nanning [38] n = 250 Survey year: 1995–1996	0	94.4	91.5	97.3	100	100.0	100.0
	1	91.2	87.7	94.7	100	100.0	100.0
	2	78.4	73.3	83.5	95.6	93.1	98.1
	3	60.8	54.7	66.9	93.2	90.1	96.3
	4	50.4	44.2	56.6	87.6	83.5	91.7

Guangzhou [39] n = 1323 Survey year: 1998–1999	0	94.0	92.7	95.2	96.4	95.4	97.4
	1	90.5	88.9	92.1	95.7	94.6	96.8
	2	83.3	81.3	85.3	93.9	92.6	95.2
	3	75.7	73.4	78.0	93.2	91.8	94.6
	4	56.6	53.9	59.3	92.8	91.4	94.2
	Total	80.0	77.8	82.2	94.4	93.2	95.6

Shenzhen [40] n = 505 Survey year: 1997.	1	66.5	62.4	70.6	76.8	73.1	80.5
	2	65.7	61.6	69.8	74.7	70.9	78.5
	3	62.0	57.8	66.2	71.5	67.6	75.4
	4	56.0	51.7	60.3	65.4	61.3	69.5

Zhuhai^c ^[25] n = 148 Survey year: 1995	0	97.3	94.7	99.9	98.6	96.7	100.5
	1	88.5	83.4	93.6	95.9	92.7	99.1
	2	81.6	75.4	87.8	95.3	91.9	98.7
	3	75.0	68.0	82.0	92.1	87.8	96.4
	4	70.9	63.6	78.2	92.1	87.8	96.4

Qiqihar [41] n = 66 Survey year: 2002.	1	63.6	52.0	75.2	72.7	62.0	83.4
	2	42.4	30.5	54.3	66.7	55.3	78.1
	3	31.8	20.6	43.0	56.1	44.1	68.1
	4	27.3	16.6	38.0	53.0	41.0	65.0

Shunde [42] n = 660 Survey year: 2000.	1	78.5	75.4	81.6	92.4	90.4	94.4
	3	69.1	65.6	72.6	86.7	84.1	89.3
	4	62.0	58.3	65.7	83.3	80.5	86.1

Luzhou [43] n = 203 Survey year: 2002	1	89.6	85.4	93.8	97.5	95.4	99.6
	2	87.7	83.2	92.2	97.1	94.8	99.4
	3	83.7	78.6	88.8	93.1	89.6	96.6
	4	79.8	74.3	85.3	90.1	86.0	94.2

Duan [44] n = 186 Survey year: 1997–1998.	0	94.1	90.7	97.5	100	100.0	100.0
	1	79.6	73.8	85.4	98.4	96.6	100.2
	2	60.8	53.8	67.8	94.1	90.7	97.5
	4	40.9	33.8	48.0	91.9	88.0	95.8

Chenyang [45] n = 149 Survey year: 2000.	1	92.6	88.4	96.8	98.7	96.9	100.5
	2	85.2	79.5	90.9	98.7	96.9	100.5
	3	80.5	74.1	86.9	98.0	95.8	100.2
	4	71.8	64.6	79.0	96.6	93.7	99.5

Shengzhou [46] n = 128 Survey year: 1997–1998.	0	96.9	93.9	99.9	100.0	100.0	100.0
	1	93.8	89.6	98.0	100.0	100.0	100.0
	2	84.4	78.1	90.7	96.9	93.9	99.9
	3	78.1	70.9	85.3	95.3	91.6	99.0
	4	76.6	69.3	83.9	91.4	86.5	96.3

Xinjiang [47] n = 578 Survey year: 2003–2004.	0	78.0	74.6	81.4	88.5	85.9	91.1
	1	33.9	29.9	37.9	85.6	82.6	88.6
	2	29.4	25.5	33.3	84.5	81.4	87.6
	3	24.0	20.3	27.7	83.1	79.9	86.3
	4	10.9	8.2	13.6	82.0	78.6	85.4

^a^: The data of Wuhan city were from two sources.

^b^: 0 month refers to the period before discharge from hospital, generally in one week after delivery or 0.5 month of age.

^c^. 'Exclusive breastfeeding' include 'full breastfeeding'

Chongqing: a large city (municipality directly under the Central Government), in Southwest China.

Wuhan, a large city (municipality directly under the Central Government), in Central China.

Nanjing, the capital city of Jangsu province, East China.

Nanning, the capital city of Guangxi province, South China.

Guangzhou, the capital city of Guangdong province, South East China.

Shenzhen, a medium-sized city in Guangdong province, Southeast China.

Zhuhai, a medium-sized city in Guangdong province, Southeast China.

Qiqihar: a medium-sized city in Heilongjiang province, Northeast China.

Shunde: a medium-sized city in Guangdong Province, Southeast China.

Luzhou: a medium-sized city in Cichuan Province, Southwest China.

Duan, a county town, Guangxi province, South China.

Chenyang, a small town in Jiangsu province, East China.

Shengzhou, a small city in Zhejiang province, East China.

Xinjiang(Han Chinese), Uygur Autonomous Region, West China.

**Table 2:** Breastfeeding rates (%) and duration in P.R. China

**Study site**	**Initiation rate (%)**	**Any breastfeeding rate at four months (%)**	**Exclusive breastfeeding rate at four months (%)**	**Breastfeeding duration (mean in months)**	**Study details**
Beijing** [24,27,53,54],	92.0 90.5	58.77	39.2 (FBF)	7.7 7.4	Cross-sectional study: study year: 1998, n = 251 study year: 1995–1998, n = 359 study year: 2002, n = 134 study year: 2002, n = 422

Shanghai** [55-57]	93.0; 97.3,	56.8; 83.3;	28.1; 41.1	7.4	Retrospective study, study year, 2002, n = 1199; Cross-sectional study, study year, 2004, n = 1877; Cohort study, study year, 1999–2000, n = 258

Chongqing** [55,58]	95.4	44.6	22.4	5.2	Retrospective study, study year, 2002; n = 383. Cross-sectional study, study year, 1999; n = 603.

Guangzhou* [39,55,59]	93.9 96.4	64.5; 92.8	50.5; 56.6	8.2	Retrospective study: study year: 2002, n = 954; study year: 1995, n = 418,; Survey year: 1998–1999, n = 1323

Fuzhou* [60]	99.9	88.7 ^b^	33.0 ^b^		Cross-sectional study, study year: 1995, n = 2972

Xi'an* [55,58,61]	95.0	70.2	37.7	9.5, 5.2(U) 8.0(R)	Retrospective study, study year, 2002; n = 491, study year, 2001, n = 9000 Cross-sectional study, study year, 1999; n = 1201.

Changchun* [55,58]	95.7	67.2	28.6	9.2	Retrospective study, study year, 2002; n = 385. Cross-sectional study, study year, 1999; n = 603

Harbin* [58,62]	71.3 (U) ^a^ 84.6 (R) ^a^	57.5 (U) 75.0 (R)	28.0		Cross-sectional study, study year: 1996, n = 603, study year, 1999; n = 596

Chengdu* [63]	92.6		54.4 ^c^		Cross-sectional study, study year, 1995–1996; n = 1527.

Taiyuan* [64]		84.1 ^c^	64.9 ^c^		Cross-sectional study, 1998–2002, n = 2140

Kunming* [65]		79.1	32.1		Cohort, 1993–1996, n = 913

Hubei ^p^ [27,31]	76 (U) ^a^, 96.6 (R) ^a^	44 (U), 91.2 (R)	60.2 (FBF)	9.8	Cross-sectional study, study year: 2002, n = 213. Retrospective study, study year: 1998, n = 2000

Guangdong^p ^[27,66]	94.9	87.2 ^b^	40.0 (FBF) 38.3 ^b^	6.3	Cross-sectional study, Study year: 2002, n = 135; Study year: 1997, n = 316

Fujian^p ^[67]	99.0	97.3 ^b^	43.1 ^b^		Cross-sectional study, study year: 1995, n = 1241

Jiangsu^p ^[68,69]	95.4 ^a^	88.0	23.1–41.65		Cohort, 1996–1997, n = 1950. Cross-sectional study, 1994–1996, n = 1065

Gansu^p ^[70],	96.0		47.6		Cross-sectional study, study year: 2001, n = 1460

Guizhou^p ^[70]	97.5		42.9		Cross-sectional study, study year: 2001, n = 1460

Zhejiang^p ^[27,71,72]	97.35(R)		52.4 (FBF); 44.0, 58.8 (R)	8.2	Cross-sectional study: study year: 2002, n = 200; study year: 1998–1999, n = 486; study year: 1996, n = 371

Shandong^p ^[27,73]	98.88		56.7 (FBF); 25.72 (FBF)	12.3	Cross-sectional study, study year: 2002, n = 220. Cross-sectional study, study year: 1996–2000, n = 21036.

Chengde^m^[74]	94.8^a^	92.9	76.3		Cohort study Study year: 1999–2002, n = 1042.

Shenzhen^m ^[75]	85.6–97.6		56.6–84.2		Cross-sectional study, study year: 1995–2000, n = 3908.

FBF: Full breastfeeding, U: urban areas, R: rural areas.

a: at one month; b: at 3–4 months; c: at 4–6 months.

p: province; m: medium-sized city.

* Capital city of province.

** Large city (municipality directly under the Central Government).

A breastfeeding education program commenced in Beijing in 1983, but breastfeeding rates remained at a low level for another 10 years. A survey undertaken in 20 provinces in China in 1984 showed that breastfeeding rates at four and six months were 42.5% and 34.4% in urban areas and 69.95% and 60.35% in rural areas [18]. In a rural area near Shanghai a survey showed the breastfeeding rate was 80% in the early 1980s and fell to 44.1% in the early 1990s [19]. The trends towards declining breastfeeding rates were similar in urban and rural areas, but the rates in urban areas were invariably lower.

To address the decline in breastfeeding, the Chinese government set a target to achieve a national 'exclusive breastfeeding' rate at four months of 80% by 2000 in the National Program of Action for Child Development in China in the early 1990s [20]. During the 1990s the Baby Friendly Hospital movement became established in China and between 1992 and 1998, Baby Friendly Hospitals were initiated in 6745 large or medium-sized hospitals and 3475 small hospitals [21]. According to Lanqin Song's report, there were 5550 small hospitals initiated as Baby Friendly Hospitals by the end of 1998 [21,22].

During this period many initiatives were commenced to promote breastfeeding, including the Baby Friendly Hospital Initiative, women and child health protection legislation, society support programs and breastfeeding education programs [21]. The breastfeeding rate in China started to increase in the 1990s [8]. In Beijing, after the Baby Friendly Hospital Initiative became established in 1992, the breastfeeding rates started to increase. After the commencement of the Baby Friendly Hospital Initiative in Longfu Hospital in Beijing, the 'any breastfeeding rate' at four months increased from 56% to 63% (from 1989 – 1992) to 83% (in 1993 – 1994); and the 'full breastfeeding' rate increased from 28% before the initiation to 40% afterwards (the cross sectional study, n = 439) [7]. Another survey in Beijing (retrospective study, n = 817) showed the breastfeeding rate at four months was only 16.42% in 1989 – 1991; but it increased to 56.73% in 1995 – 1997 [23]. A survey at the Beijing Railway Hospital (retrospective study, n = 824) showed the same trend: the breastfeeding rate at four months was 16.77% in 1991 – 1994 and 58.77% in 1995 – 1998 [24].

The trends in breastfeeding in other areas of China were similar to those that occurred in Beijing [19]. In Tianjin, one of the large cities in eastern China, a major breastfeeding promotion program started in 1987. The breastfeeding initiation rates in Maternal and Child Health Care Institute of Tanggui District in the city increased from 55% in 1985 to 85% in 1991 and 95% in 1992 (cross sectional study, n = 1897 in 1992, although the sample sizes were not given in the previous results) [8].

A cohort study (n = 289) in Xiangzhou People's Hospital in Zhuhai of Guangdong province showed the 'full breastfeeding' rates at four and six months were 45.4% and 9.9% respectively in 1993 [25]. After the Baby Friendly Hospital Initiative was introduced in the hospital the 'full breastfeeding' rates at four and six months were 70.9% and 56.8% respectively in 1994 [25].

A national cross sectional survey in 1992 (n = 177163) showed that 'full breastfeeding' rates after the first month were 21.1% in urban areas and 37.6% in rural areas, and at three months were 14.3% and 24.3% respectively [26]. In 1998 another national cross sectional survey (n = 13721) showed an increase in the 'full breastfeeding' rate: 'full breastfeeding' rates at one month were 64.3% in urban areas and 60.0% in rural areas and at three months 37.5% and 53.7% respectively [26]. In 2002, a cross-sectional study (n = 2001) undertaken in Beijing and four provinces (Shandong, Hubei, Zhejiang, Guangdong) found that 'ever breastfed' rate was 90.1% and the 'full breastfeeding' rates at four and six months were 45.3% and 21.6% respectively[27]. The survey also documented that the average duration of breastfeeding was 8.73 ± 4.21 months (mean ± SD) [27]. A retrospective survey (n = 3414) in five large cities from different regions of China (Guangzhou, Shanghai, Chongqing, Xi'an and Changchun) in 2002 showed that average breastfeeding duration was eight months and the 'any breastfeeding' rates at 0, 4, 6, 12, 24 months were 94.6%, 61.0%, 50.1%, 5.3% and 0.4% respectively [28].

A major survey in 105 rural counties from across the country (sample size = 21036) from 1996 to 2000 found that 'ever breastfeeding' rate was 98.22% and 'full breastfeeding rate' at four months was 24.35% in these rural areas [29]. A comparison of breastfeeding rates made in Shihezi, a remote area in Xinjiang Province in the far west of China, showed that there was no significant increase in 'any breastfeeding' rates at six months (77.5% in 1994 – 1996, 76.2% in 2003 – 2004) but the 'full breastfeeding' rate at one month increased from 38.0% in 1994 – 1996 to 57.3% in 2002 – 2004 (p < 0.05) [30]. The results analyzed from data of Chinese Food and Nutrition Surveillance System (CFNSS) in 1998 (cross sectional study, n = 403) showed that the 'exclusive breastfeeding' rates under 4 months were 67.1% (53.7% in urban areas, 76.6% in general rural areas, and 64.6% in the poor rural areas) [26].

Collectively these studies show that the breastfeeding rates in China fell during the 1970s, reaching a nadir in the 1980s and then began to rise in the 1990s. However China is a huge country, geographically as well as in population and ethnic diversity, and breastfeeding rates in different parts of China can vary considerably [31,32].

These studies also show that breastfeeding in China has significantly improved since the introduction of the Baby Friendly Hospital Initiative beginning in 1992. Before the implementation of breastfeeding promotion programs, few studies of breastfeeding were published. However since 1993, there has been a notable increase in the number of published studies on breastfeeding. In the next section the results of studies that have included breastfeeding rates from different regions of China undertaken in the past decade are discussed.

### Breastfeeding rates in China from cohort studies

Table 1 summarises 'exclusive breastfeeding' and 'any breastfeeding' at four months in six large, five medium-sized and three small cities and a region in China. The cities and regions included in this table are Beijing [33], Chongqing [34], Wuhan [35,36], Nanjing [37], Nanning [38], Guangzhou[39], Shenzhen [40], Zhuhai [25], Qiqihar [41], Shunde [42], Luzhou [43], Duan [44], Chenyang [45], Shengzhou [46] and Xinjiang [47]. These results are from 16 papers published in Chinese and English describing breastfeeding in cohort studies from 1994 to 2004.

**Figure 2:**
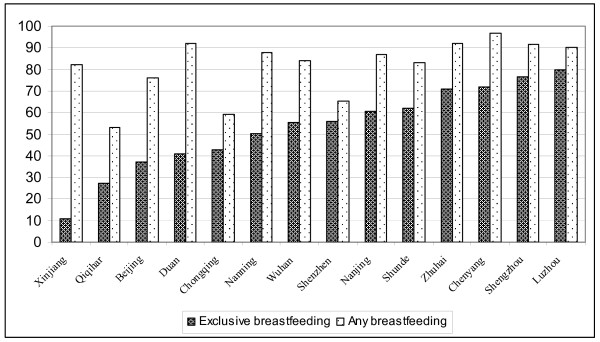
**Breastfeeding rates (%) at four months from cohort studies, P.R. China**.

Criteria for the review in Table 1 were cohort studies; followed up monthly at least to four months; variables included 'exclusive breastfeeding', 'Partial Breastfeeding' and non-breastfeeding; and that the research was from China. In Table 1, 'any breastfeeding' rates were generated from 'exclusive breastfeeding' plus 'Partial Breastfeeding'. Figure 2 shows the breastfeeding rates (%) at four months from cohort studies.

The methods used in these studies were similar. All cases were recruited randomly in hospitals after delivery. The follow-up data were consecutive and collected by medical professionals at their postpartum visit or babies' regular physical examination. Most cases (93%) completed follow-up to at least four months.

Table 1 shows that 'any breastfeeding' rates at four months were very different in the six large cities: 93% in Guangzhou, 84% to 88% in Nanning, Nanjing and Wuhan, 76% in Beijing, but only 59% in Chongqing. Similarly, the rates ranged from 53% to 92% in the four medium-sized cities. The rates were all above 90% in the three small cities and 82% in Xinjiang Uygur Autonomous Region.

The 'exclusive breastfeeding' rate in all cities studied except Luzhou was below 80% at four months after birth. The reported rates of 'exclusive breastfeeding' ranged from 11% to 80%. In the six large cities, 'exclusive breastfeeding' rates at four months ranged from 37% to 61%; in the five medium-sized cities, from 27% to 80%; in the three small cities, from 41% to 77% and in Xinjiang Uygur Autonomous Region was only 10.9%. Both 'any breastfeeding' and 'exclusive breastfeeding' rates in Chongqing and Qiqihar were relatively lower than the majority of cities. 'Exclusive breastfeeding' rates in Beijing and Duan were also lower, but the lowest was in Xinjiang.

There were other cohort studies which did not have monthly follow-up or the variables studied were not as detailed as those in Table 1. A cohort study in a Shanghai suburb showed that 'any breastfeeding' rates at four months were above 90% from 1994 to 1996 [48]. 'Exclusive breastfeeding' rates at one month were 48%, 60% and 80% in 1994, 1995 and 1996 respectively and at four months 20%, 36% and 70% respectively [48]. Thanks to breastfeeding promotion, the 'exclusive breastfeeding' rates increased over the years. But 'exclusive breastfeeding' has not yet reached 80%. A cohort study (n = 1085) in Daqing, a medium-sized city in Heilongjiang Province, showed that 'full breastfeeding' rates at one, two, three and four months were 86.5%, 82.6%, 75.8% and 71.2% respectively [49].

A cohort study in Nanchang, the capital city of Jiangxi province showed that 'exclusive breastfeeding' was 67.7% at one month and 45.8% at four months [50]. In Nantong, a medium-sized city in Jiangsu province, the 'exclusive breastfeeding' rates at discharge and at four months were 81.63% and 58.16% respectively [51]. Another cohort study in Chongqing in 2003 showed that 'exclusive breastfeeding' rates at one, two, three and four months were 98%, 69%, 66% and 64% respectively [52]. The 'exclusive breastfeeding' rates were higher than in 1999 [34].

Overall, the reported 'any breastfeeding' rates in the majority of cities were above 80% at four months in these cohort studies. However most cities did not reach the national target of 'exclusive breastfeeding' of 80%.

### Breastfeeding rates in China from other studies

**Figure 3:**
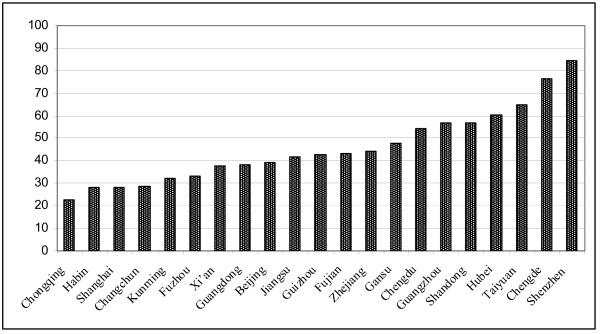
**Exclusive breastfeeding rates (%) at four months from cross-sectional or retrospective studies in P.R. China**. (For those provinces or cities with more than one study, the latest available results are shown in Figure 3.

Table 2 summarises 'exclusive breastfeeding' and 'any breastfeeding' rates at four months, breastfeeding initiation rates and duration in eleven large cities, eight provinces and two medium-sized cities in China. The cities and provinces included in Table 2 are Beijing [24,27,53,54], Shanghai [55-57], Chongqing [55,58], Guangzhou[39,55,59], Fuzhou [60], Xi'an [55,58,61], Changchun[55,58], Harbin[58,62], Chengdu[63], Taiyuan [64], Kunming[65], Hubei [27,31], Guangdong [27,66], Fujian [67], Jiangsu [68,69], Gansu [70], Guizhou [70], Zhejiang [27,71,72]; Shandong [27,73], Chengde [74]and Shenzhen [75]. Some of these results are also shown in Figure 3, where exclusive breastfeeding rates (%) at four months from cross-sectional and retrospective studies are shown. Some of these results are also shown in Figure 3, where exclusive breastfeeding rates (%) at four months from cross-sectional and retrospective studies are shown. For those provinces or cities where there was more than one study, the latest results are presented. The research methods included cross-sectional, retrospective studies and some cohort studies which did not satisfy the criteria for Table 1.

The national surveys (cross-sectional) showed that 'exclusive breastfeeding' rates at three months were 46.3% in urban areas and 69.6% in rural areas, and at four months 24.4% [58,76]. The Chinese National Nutrition and Health Survey in 2002 (cross-sectional study, n = 6858) showed a 'full breastfeeding' rate under four months of 71.6% (65.5% in urban areas and 74.4% in rural areas) and the 'any breastfeeding' rate was 94.6% (92.5% in urban areas and 95.6% in rural areas) [77]. In the survey, the 'any breastfeeding' rate at four months was 91.2%, at six months 84.3%, at one year 42.6% and at two years 19.2%. The mean duration of breastfeeding was 10.1 months (8.6 months in urban areas and 10.8 months in rural areas) and the complementary food introduction rate before four months was 30.4% [77]. A cross-sectional survey from Hefei showed the 'exclusive breastfeeding' rate at 4–6 months was 51.4% [78].

In eight of the large cities out of eleven shown in Table 2, the 'exclusive breastfeeding' rates at four months were below 40%. In the eight provinces shown in Table 2, the 'exclusive breastfeeding' rates at four months ranged from 40% to 60%. The only report in which 'exclusive breastfeeding' at four months reached 80% in Table 2 was from Guanlan Hospital in Shenzhen, a medium-sized city in Guangdong Province and close to Hong Kong. In this hospital, 'exclusive breastfeeding' has increased significantly since the Baby Friendly Hospital Initiative was introduced in 1995. The 'exclusive breastfeeding' rates were 63.4% in 1995, 56.6% in 1996, 63.8% in 1997, 73.3% in 1998, 78.6% in 1999 and reached 84.2% in 2000 [75].

Similar to cohort studies, 'any breastfeeding' rates in the majority of cities or provinces were above 80% at four months. But almost all cities or provinces did not reach the national target of 'exclusive breastfeeding' of 80%. The durations of 'any breastfeeding' in the majority of cities or provinces were between seven and nine months.

### Breastfeeding in minority areas or groups

In Xinjiang Uygur Autonomous Region, the 'exclusive breastfeeding' rate at four months was 19. 4% (in 2000) [70]. A survey in Shihezi showed 'exclusive breastfeeding' rates in the first week of life were 53% in Uygur, 62% in Kazakh, 33% in Hui and 41% in Han ethnic groups. Overall the 'exclusive breastfeeding' rate in the first four months was 29.7% (1993 – 1995) [79]. Another survey in Shihezi in Han ethnic people showed that 'any breastfeeding' rates were 82% at four months in both 1994 – 1996 and 2003 – 2004, 78% at six months in 1994 – 1996 and 76% at six months in 2003 – 2004; the 'exclusive breastfeeding' rates at four months were 25% in 1994 – 1996 and 10% in 2003–2004 and the rates at six months were 9% and 4% in 1994 – 1996 and 2003 – 2004 respectively [30]. A survey in Uygur group in Xinjiang (in 2003 – 2004) showed that 'any breastfeeding' rates were 84.8% at four months and 54.7% and six months, 'exclusive breastfeeding' rates were 1.9% and 0.4% at four and six months respectively [47] A survey from Karamay (1996–1997), a medium city in Xinjiang showed an 'any breastfeeding' rate at four months of 95.9% and 'exclusive breastfeeding' rate of 64. 9%. The mean introduction time of solid food was 5 months (4. 7 months in the majority Han group and 5. 7 months in minority groups) [80].

In the Tibet Autonomous Region, 'any breastfeeding' rates from one month to 4–6 months were 89% to 83% in urban areas, and 93% to 90% respectively in rural areas in 1999. But the 'full breastfeeding' rate in this region was very low. The 'full breastfeeding' rates in urban areas were 25% at one month, declining to 3% at 4 – 6 months. In rural areas, the 'full breastfeeding' rates were 37% at one month, declining to 13% at 4 – 6 months. The average breastfeeding duration in Tibet children was 14.2 months, the longest duration in China[81].

In Yunnan province, a survey in a rural area showed that the breastfeeding initiation rate was 95% in both 1991 and 1994. The 'any breastfeeding ' rates at four months were 81.6% in 1991 and 66.2% in 1994 [82]. Another cross-sectional study in Yunnan province found that the 'any breastfeeding' rate at four months was 93.6%, the 'full breastfeeding' rate 43.0% and 'exclusive breastfeeding' 35.1% in 2001 – 2002 [83].

In Inner Mongolia, a retrospective study in Baotou city in 1999 showed that 'exclusive breastfeeding' rates were 83.8% at discharge, 56.1% at one month and 50.0% at four months[84].

In Ningxia Hui Autonomous Region, the 'any breastfeeding' rate at 2 weeks was 98.4% and the 'exclusive breastfeeding' rate at four months was 36. 8% in 2001 [70].

In Qinghai province, the 'any breastfeeding' rate at 2 weeks was 93.8% and the 'exclusive breastfeeding' rate at four months was 39.2% in 1999 [70].

A survey in Yanji city, Jilin Province from 1993 to 1994 showed that the 'any breastfeeding' rate at four months was 73.36% in the Korean ethnic and 79.58% in the Han ethnic groups. The difference was not significant [85].

In the minority areas, three studies out of four showed that breastfeeding initiation rates were above 90% and the rest above 84%. The 'any breastfeeding' rates at four months were 83–90% in Tibet, and 66–94% in Yunnan. The 'exclusive breastfeeding' rates at four months were 10–25% in Xinjiang, 35% in Yunan, 37% in Ningxia, 39% in Qinghai and 50% in Inner Mongolia. In Tibet, the 'exclusive breastfeeding' rates at four months were below 13%, the lowest in China although the rate in the ethnic groups of Xinjiang Province was almost as low. The 'exclusive breastfeeding' rates in minority areas were lower than inland provinces.

### Reasons for discontinuing breastfeeding or exclusive breastfeeding before four months in China

Table 3 shows the reasons for ceasing breastfeeding or introducing water or other infant food before four months reported in these studies. The cities and regions for which reports were available were Beijing[23,24], Shanghai [57,86,87], Kunming [65], Zhejiang rural area [72], Jiangsu [68] and Sichuan [88]. The common reasons for ceasing breastfeeding or 'exclusive breastfeeding' before four months were: perceived breast milk insufficiency, mother going to work, maternal and child illness and breast problems.

**Table 3:** Reasons for discontinuing breastfeeding or exclusive breastfeeding before four months

**Research site**	**Breast milk 'insufficient' (%)**	**Mother returning to work (%)**	**Maternal or child illness (%)**	**Breast problems (%)**	**Mother dislikes or feels uncomfortable with breastfeeding (%)**	**Other reasons (%)**
Beijing** (n = 397, 1989–1991, [23])	8.3	13.6	18.4		32.2	27.50

Beijing** (n = 387, 1991–1994, [23])	8.8	19.4	14.0	6.9	35.0	15.90

Beijing** (n = 148, 1995–1997, [24])	34.5	25.0	19.6	4.1	13.5	3.30

Beijing** (n = 148, 1995–1998, [24])	47.3	20.4	16.2	5.4		10.70

Shanghai* (n = 152, [57])	49.3	21.7	15.8	3.9		9.30

Shanghai* (n = 131, [86])	41.2	37.4	9.1		9.9	2.40

Shanghai**(n = 86, [86])	59.3	15.1	15.1	1.2		9.30

Kunming* (n = 140, [65])	48.6	4.3	4.3		5.0	37.80

Zhejiang** rural area (n = 153, 1996, [72])	52.9	5.2	9.1	6.5		26.30

Jiangsu** (n = 235, 1996–1997, [68])	41.3	28.1	22.6			8.00

Sichuan** (n = 148, 2001, [88])	13.5		31.1	24.3		31.10

* Reasons for discontinuation of 'exclusive breastfeeding'

** Reasons for discontinuation of 'any breastfeeding'

### Perceived breast milk insufficiency

Perceived breast milk insufficiency was the most common reason for discontinuing 'exclusive breastfeeding' or 'any breastfeeding' in China [61,89,90]. For example, in Tibet, perceived breast milk deficiency was the main reason for weaning both in urban and rural areas [81]. In Xi'an, 'insufficient milk' was the first reason (81%) for terminating breastfeeding [61]. A survey in Hubei province showed that in mothers who gave their babies complementary food before four months, 51.7% of them thought their breast milk supply was insufficient [31]. These results are consistent with studies in other countries [91]. A study in Japanese women living in Perth, Australia, showed that the most common reason for the decision to cease breastfeeding was 'insufficient breast milk' [92]. The perceived 'insufficient milk' may not reflect the true reasons for cessation of breastfeeding but may be given by mothers as a socially acceptable reason when she wanted to stop breastfeeding [91]. The study in Beijing (Table 3) showed that before the Baby Friendly Hospital Initiative (before 1995), one third of mothers (32 – 35%) claimed that they terminated breastfeeding because of disliking or feeling uncomfortable with breastfeeding. Only 8–9% mothers ceased breastfeeding because of perceived insufficient milk. But after the introduction of the Baby Friendly Hospital Initiative, mothers were more aware of the importance of breastfeeding their babies and less than 14% mothers gave the reason for stopping breastfeeding as 'dislike or feel uncomfortable with breastfeeding'. The reason of 'insufficient milk' increased to more than 35% while other reasons changed little [23,24]. It appeared that mothers changed the excuse of 'dislike' to 'insufficient milk' for not breastfeeding their babies. In a study of 214 mothers in Kunming, 68 mothers introduced complementary food before four months and 66% of them claimed the reasons as 'insufficient milk' or 'no milk'. Among the mothers who claimed 'breast milk insufficiency', 28 mothers thought they had temporary 'insufficient milk' and fed their babies infant formula. Once formula was introduced, their breast milk production decreased. Thirteen babies could not suck at the breast because of the mother's operative pain or early birth. Nine mothers suspected that their breast milk quality was not good enough and seven mothers reported having had insufficient sleep. Only 11 mothers (5%) claimed that breast milk insufficiency was the only reason [65].

### Mothers returning to work

In metropolitan cities or developed areas, the mother returning to paid employment was an important reason for breastfeeding cessation or early introduction of complementary food [31,61,89]. Maternity leave in China included three months delivery leave and one month lactation leave for working mothers[16]. The total of four months leave was not enough for 'exclusive breastfeeding' for six months [66,80]. In some companies, maternity leave was less than four months. In Zhongshan City of Guangdong province, the average delivery leave was only 67 days and only 2.6% of work places had breastfeeding rooms [66].

### Mother or child illness

Maternal or child health problems were one of the main reasons for stopping breastfeeding or introducing complementary food [61,89]. In Beijing, illness of the mother or baby accounted for 14 – 20% of reasons (rank third) for terminating breastfeeding[23,24]. This reason ranked second for ceasing breastfeeding and third for early complementary food introduction in Shanghai [57,86,87]. It was ranked third in Jiangsu [68], second in Kunming [65] and Zhejiang [72], and first in Sichuan province [88]. Maternal illness such as breast inflammation or hepatitis B (HBsAg positive) often led to separation from babies and cessation of breastfeeding [88]. In the infant group, the main health problems were respiratory diseases and diarrhea [57,61]. Premature or low birth weight infants often stayed in an Intensive Care Unit for a period of time and were less likely to be breastfed [88]. Many maternal or infant health problems could be overcome with good health care and appropriate advice and should not be the reasons for stopping breastfeeding.

### Breast problems

Breast problems such as sore and inverted nipples, and mastitis were very common in breastfeeding mothers especially in the first month [57,87,88].

### Incorrect traditional perceptions

The tradition of introducing complementary food early to infants can be traced as far back as the Sui Dynasty (581 – 618AD) of China [93]. An academic report from this period stated: 'thirty days after a child is born, he should be given some foods, in the amount of two dates or so; after fifty days, of a cherry or two; after a hundred days, of a large date or so'. Tang (618 – 907AD) medical authorities were quoted: 'an infant may be given a rice drink after the seven days of birth'. And a scholar named Sheng Chi Ching in Song Dynasty (960 – 1276AD) also advised that foods should be provided to an infant from thirty days after birth [93].

Even today, these incorrect traditional perceptions still have a strong adverse influence on 'exclusive breastfeeding' in China, especially in less developed and minority areas [65,72]. The Chinese National Nutrition and Health Survey in 2002 (n = 6858) found that the first feed of 48.8% babies (38.8% in urban areas and 53.1% in rural areas) was sugar water [77]. Following traditional feeding methods was an important reason for discontinuing 'exclusive breastfeeding' in Kunming (accounted for 34% of reasons) [65] and Zhejiang rural areas (26% reasons) [72].

In Hubei province, the most common reason for the introduction of complementary food before four months for half of all women, was that the mother wanted to follow traditional feeding practices [31]. Commonly, mothers thought that their breast milk was 'too thin' to satisfy their baby's requirement and they needed to add cereal to complement breast milk [60]. Traditional feeding methods have had a strong influence on infant feeding practices in China. One medical professional misunderstood 'exclusive breastfeeding' as 'do not give baby formula or cow's milk in five months, but can give some starchy food' [82]. Many mothers thought that the baby should be weaned (ie. discontinue breastfeeding) before 12 months [81].

Another traditional perception which needs to be corrected is that 'breastfeeding is not good for mothers'. A survey conducted at the Beijing Women Hospital from 1983 to 1985 showed that almost half of mothers and relatives thought breastfeeding was not acceptable because it 'increased a mother's burden', impacted on mother's health or changed the mother's shape [15].

### Other reasons

Caesarean section rates have increased significantly in China since the mid-1990s [29]. The caesarean section rate in China was reported as being 1 – 2% in 1950s, 4 – 6% in 1960s, rising to be more than 20% in 1980s [29]. More recently rates of caesarean section in China have risen rapidly to reach as high as 77% in Hangzhou [94]. Cesarean section has become an important reason for discontinuing breastfeeding in Sichuan (accounted for 16% reasons) [88] and Beijing (accounted for 5% reasons)[23,24]. A cohort study in Shanghai showed that breastfeeding rates at 1, 6 and 12 months in the caesarean section group were significantly lower than vaginal delivery group with a Hazard Ratio of 1.21 (95%CI: 1.10, 1.33)[95]. In rural areas of Tibet, maternal pregnancy was an important reason for stopping breastfeeding [81].

There are several limitations for this review. We have endeavoured to find all relevant studies. However there is only one extensive electronic data base in the Chinese language. There may be some small regional journals that are not included in this database. Extensive travel throughout regional China would be required to ascertain whether these studies even exist. We have relied on the databases, our own knowledge of the field and consultations with colleagues. The study does not include several special areas of China, namely Macao, Hong Kong and Taiwan. Any studies published in languages other than English or Chinese have not been included.

As a next stage in this research we would recommend that studies should be undertaken in the major medical libraries in each Province. This would require the services of experienced Chinese librarians and extensive travel and consultations.

## Conclusion

Breastfeeding rates in China fell during the 1970s when the use of breastmilk substitutes became widespread. Breastfeeding reached its lowest point in the 1980s. As a result many efforts were introduced to promote breastfeeding, and the breastfeeding rate in China started to increase in the 1990s. Since the mid-1990s 'any breastfeeding' rates in the majority of cities and provinces including minority areas were above 80% at four months. But very few cities and provinces reached the national target for 'exclusive breastfeeding' of 80%. The mean duration of 'any breastfeeding' in the majority of cities or provinces was between seven and nine months.

## Competing interests

The authors declare that they have no competing interests.

## Authors' contributions

XF translated literature and wrote manuscript. LQ wrote manuscript. CWB wrote and revised manuscript. XL wrote and revised manuscript.
